# Real-world use of oral versus subcutaneous semaglutide in a cohort of type 2 diabetic patients: which option to which patient?

**DOI:** 10.1007/s40618-024-02369-4

**Published:** 2024-04-29

**Authors:** C. Formichi, W. Baronti, G. de Gennaro, M. Cerrai Ceroni, L. Nigi, L. Rizzo, F. Dotta

**Affiliations:** 1 Diabetes and Metabolic Diseases Unit, Department of Medicine, Surgery and Neurosciences, University of Siena, 53100 Siena, Italy; 2 Fondazione Umberto Di Mario, Toscana Life Science, 53100 Siena, Italy; 3 Metabolic Diseases and Diabetes Unit, Misericordia Hospital, USL Toscana sud est, 58100 Grosseto, Italy

**Keywords:** Type 2 diabetes, GLP1-RA, Semaglutide, Tailored treatment

## Abstract

**Purpose:**

To evaluate the variables influencing the therapeutic choice toward oral versus subcutaneous semaglutide in a cohort of diabetic subjects.

**Methods:**

We retrospectively collected data of 292 patients followed at the Diabetes Unit of the University Hospital of Siena and the Hospital of Grosseto, who were prescribed oral (n = 115) or subcutaneous (n = 177) semaglutide between October 2021 and October 2022.

**Results:**

Oral semaglutide was preferentially prescribed in older subjects with longer disease duration in replacement of other antidiabetic drugs, while subcutaneous semaglutide was preferentially prescribed in add-on to metformin in subjects with higher body weight and BMI. After 6 months, both formulations significantly improved glycemic control and body weight, however injectable semaglutide showed a greater efficacy on A1c levels, weight loss, BMI and waist circumference reduction. No differences were found in terms of adverse events.

**Conclusion:**

In our experience, injectable semaglutide is preferred in patients with excess weight and shorter disease duration, while the oral formulation was used later and especially after therapeutic failure of previous therapies. Follow-up data indicate similar tolerability and efficacy of both formulations, despite subcutaneous semaglutide demonstrated greater efficacy.

**Supplementary Information:**

The online version contains supplementary material available at 10.1007/s40618-024-02369-4.

## Introduction

Glucagon-like peptide-1 receptor agonists (GLP1-RAs) are an effective class of drugs for the treatment of type 2 diabetes mellitus (T2D), with well-defined safety and tolerability profiles. Their use is associated with better glycemic control and a low risk of hypoglycaemic events in patients for whom other oral antidiabetic drugs (OADs), such as metformin, are not sufficient to achieve adequate glycemic control [1]. Moreover, several specifically designed randomized clinical trials demonstrated a significant reduction in the incidence of cardiovascular events (CV) with some of these agents [2–4]. Therefore, the national and international guidelines recommend GLP1-RA as first-line therapy for adults with T2D with established cardiovascular disease [5, 6].

Among GLP1-Ras, semaglutide is currently the only drug available in both subcutaneous and oral formulation. Subcutaneous semaglutide is a long-acting GLP1-RA once-weekly extended release. It has been investigated in the Semaglutide Unabated Sustainability in Treatment of Type 2 Diabetes (SUSTAIN) program, which showed significant reductions in glycated hemoglobin (A1c) and body weight (BW) compared to placebo or other treatments [7–14]. Furthermore, a significant reduction in major adverse cardiovascular events (MACE) rates was observed with SC semaglutide compared to placebo [15].

Oral semaglutide is the first GLP1-RA developed for oral administration and it was approved for the treatment of T2D in adults by the European Medicines Agency (EMA) and Food and Drug Administration (FDA) in 2020 [16]. Its efficacy, in terms of improving glycemic control and BW, and safety has been demonstrated in the Peptide Innovation for Early Diabetes Treatment (PIONEER) study program [17–20]. Moreover, oral semaglutide has been proved non-inferior to placebo in terms of CV safety [21], providing a new choice for the management of T2D and a convenient administration route for patients who prefer oral treatments over injectable therapies.

Nevertheless, complementary real-world evidence is needed to further understand and support clinical decision-making in preferring oral or subcutaneous formula. Here, we show the results of our retrospective study, designed to investigate the presence or absence of different prescribing strategies between oral semaglutide and subcutaneous semaglutide in a real-world clinical setting. As a secondary endpoint, we assessed the efficacy and tolerability of the two formulations in our cohort of subjects.

## Materials and methods

### Study population

The study recruited 292 T2D patients, followed in the Diabetes and Metabolic Disease Unit of the University Hospital of Siena (Italy) and the Diabetic Unit of the Hospital of Grosseto (Italy). All patients who met the current criteria for GLP1-RA treatment and were prescribed with semaglutide, either in the oral or injective formulation, from October 2021 to October 2022, were enrolled in the study.

All clinical visits and therapy modifications were conducted according to good clinical practice. During baseline visit, we assessed clinical (i.e. blood pressure) and anthropometric measures, including BW, height, waist circumference (WC) and calculation of body mass index (BMI), and collected biochemical data, including A1c, fasting plasma glucose (FPG), renal and hepatic function and lipid profile.

After initial assessment, 115 patients (67 males/48 females, mean age ± SD 65.2 ± 10.4 years, range 35–86) were selected to receive oral semaglutide and 177 patients (105 males/72 females, mean age ± SD 62.4 ± 10.0 years, range 27–87) were prescribed once-weekly subcutaneous semaglutide. Patients were instructed to titrate semaglutide according to label information: oral semaglutide was started at 3 mg once daily (OD) and up-titrated to 7 mg OD after 30 days, unless differently required; subcutaneous semaglutide was initiated at 0.25 mg once-weekly (OW) for 4 weeks and then up-titrated to 0.5 mg OW. If clinically indicated, semaglutide dose could be further increased to 14 mg orally once daily or 1 mg subcutaneously once weekly. Patients were also instructed to take semaglutide tablets fasting, with no more than half a glass of water and wait 30 min before eating or drinking. Patients were evaluated before (T0) and after six months (T6) from GLP1-RA initiation. At follow-up visit, treatment efficacy was evaluated through clinical examination and routine blood exams. Information on side effects, self-monitoring blood glucose and treatment compliance were also collected.

### Statistics

Statistical analysis was performed with GraphPad Prism vers. 8.1.1. (GraphPad Prism, La Jolla, CA, USA). Paired t-test was used to analyze the differences between baseline and T6 parameters. Non parametric Mann—Whitney U test were used to determine the differences between groups. To compare variables among categories, the Fisher's exact test or Chi-square test were used. A *p*-value < 0.05 was considered significant.

## Results

### Baseline characteristics seem to determine the treatment choice

Patient characteristics are shown in Table 1. Patients in the two groups were homogeneous in terms of male/female ratio. Patients in the oral semaglutide group showed a significantly longer disease duration (*p* = 0.0004) and older age (*p* = 0.008) than subcutaneous semaglutide group (Fig.S1a, b), while patients prescribed with OW subcutaneous semaglutide had higher BMI and BW compared to the oral semaglutide group (*p* < 0.0001 for both parameters) (Fig.S1c, d). Consistently with the presence of obesity, patients in the subcutaneous semaglutide group also showed higher WC (*p* = 0.0002) and systolic blood pressure (*p* = 0.033), but similar diastolic blood pressure (Fig.S1e–g).

**Table 1:** Anthropometric measures and metabolic data of diabetic patients at baseline prescribed with oral or subcutaneous semaglutide

	Oral Semaglutide (n = 115)	OW Semaglutide (n = 177)	*p* value
Age (years)	65.2 ± 10.4 (35–86)	62.4 ± 10.0 (27–87)	**0.008**
M/F	67/48	105/72	0.38
Smokers/non-smokers	49/19	58/32	0.06
Very high/high CV risk	52/45	82/56	0.4
Disease duration (years)	12.8 ± 10.2 (0.2–57)	8.8 ± 8.8 (0.0–44)	**0.0004**
BW (kg)	80.0 ± 15.2 (49.5–134)	100.2 ± 22.8 (50.0–166)	** < 0.0001**
BMI (kg/m^2^)	29.1 ± 4.7 (20.3–44.1)	35,2 ± 7,3 (21.5–61.7)	** < 0.0001**
WC	106.7 ± 13.9 (90–153)	120.6 ± 16.1 (93–155)	**0.0002**
A1c (%)	7.7 ± 1.3 (5.3–12.2)	7.7 ± 1.3 (5.4–12.4)	0.94
FPG (mg/dL)	146.3 ± 38.7 (70–282)	151.3 ± 43.8 (81–344)	0.44
Total cholesterol (mg/dL)	175.1 ± 42.1 (88–295)	179.8 ± 46.6 (93–352)	0.52
Triglycerides (mg/dL)	157.1 ± 80.6 (53–545)	162.0 ± 114.4 (50–1198)	0.91
HDL (mg/dL)	47.3 ± 11.6 (22–74)	47.7 ± 12.8 (20–94)	0.96
LDL (mg/dL)	99.3 ± 38.1 (26–216)	100.2 ± 38.3 (27.2–220)	0.94
Creatinine (mg/dL)	0.95 ± 0.3 (0.47–1.86)	0.92 ± 0.25 (0.50–2.22)	0.8
eGFR (mL/min/1.73 m^2^)	78.3 ± 21.6 (34.0–118.7)	81.5 ± 20.2 (21.0–118.5)	0.3
Albuminuria (mg/dL)	30.1 ± 99.1 (0–700)	43.1 ± 107.4 (0–900)	**0.0028**
ACR (mg/g)	4.1 ± 7.02 (0.28–28.3)	26.4 ± 44.9 (0–149.8)	0.23
AST (UI/L)	20.3 ± 8.6 (10–58)	22.3 ± 11.3 (6–74)	0.37
ALT (UI/L)	25.4 ± 18.8 (8–98)	30.2 ± 21.5 (5–127)	0.12
GGT (UI/L)	26.3 ± 18.3 (6–95)	43.5 ± 47.6 (6–317)	**0.01**
Uric acid	5.5 ± 1.2 (2.8–8)	5.8 ± 1.4 (2.9–9.7)	0.31
SBP	137.2 ± 17.6 (95–180)	144.6 ± 21.0 (105–200)	**0.033**
DBP	80.0 ± 10.0 (60–100)	81.5 ± 12.2 (50–114)	0.35

Data are expressed as mean ± SD, range is shown in brackets. *M/F* male/female; *BMI* body mass index; *BW* body weight; *WC* waist circumference; A1c: glycated hemoglobin; *FPG* fasting plasma glucose; *HDL* high density lipoprotein; *LDL* low density lipoprotein; *eGFR* estimated glomerular filtration rate, calculated by MDRD (Modification of diet in renal disease) equation; *ACR* albumin-to-creatinine ratio; alanine transaminase (ALT); aspartate transaminase (AST); gamma-glutamyl transpeptidase (GGT); *SBP* systolic blood pressure; *DBP *diastolic blood pressure. Statistics using non-parametric Mann–Whitney U test and Fisher’s exact test

Baseline glycemic control, lipid profile and other metabolic parameters (renal and liver function) were similar between the two groups, except for higher levels of gamma-glutamyl transpeptidase (GGT) (*p* = 0.01) in the subcutaneous semaglutide group, possibly related to hepatic steatosis (Fig.S1h–u).

We did not find significant differences in the rate of micro- and macrovascular complications between the two groups except for a slightly—though significant—higher prevalence of carotid atherosclerosis in patient prescribed oral semaglutide (*p* = 0.013) (data not shown). The number of smokers and non-smokers (16.5% vs 42.6% in the oral and 18.1% vs 32.7% in the subcutaneous semaglutide group, respectively) was similar between the two groups. Both formulations were mainly prescribed in patient at high (39.1% in oral and 31.6% in subcutaneous semaglutide group, respectively) or very high (45.2% in oral and 46.3% in subcutaneous semaglutide group, respectively) cardiovascular risk, without significant differences between the two groups (Supplementary Fig.S1v).

### Treatment schedule

**Fig. 1:**
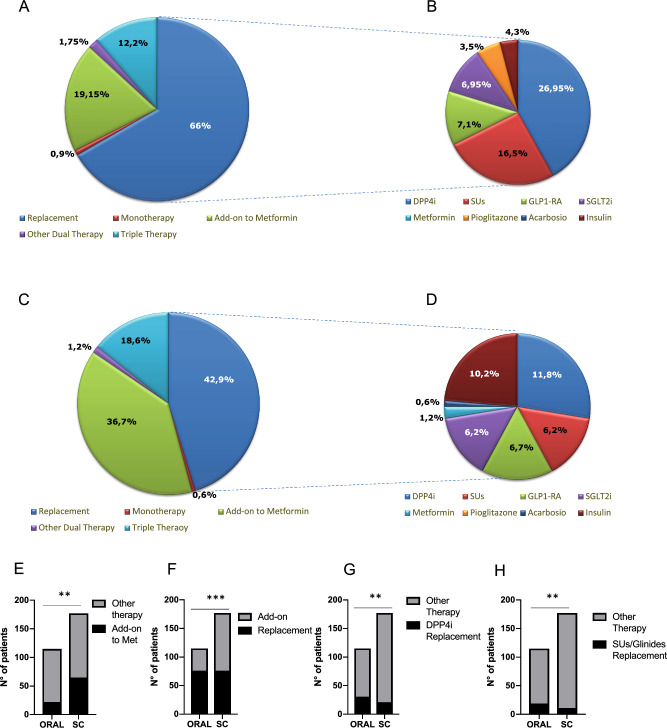
Graphical representation of therapeutic regimens prescribed in the oral **(A)** and subcutaneous **(C)** semaglutide group and detail of previous oral or injectable treatments replaced by oral **(B)** or subcutaneous **(D)** semaglutide. Comparison of the prescription rate of oral and subcutaneous semaglutide in addition to metformin **(E)**, in replacement of previous treatment **(F)**, in replacement of previous DPP4-I **(H)** or previous sulfonylureas or glinides **(I)**. DPP4i: Dipeptidyl peptidase-4 inhibitor; GLP1: glucagon-like peptide 1; SGLT2i: sodium-glucose cotransporter 2 inhibitor; SUs: sulphonylureas. *p < 0.05, **p < 0.01, ***p < 0.0001 by Fisher’s exact test

When investigating the treatment regimen more frequently used in both groups, OW subcutaneous semaglutide was more commonly prescribed as first-step intensification therapy in add-on to metformin compared to oral semaglutide (36.7% versus 19.15% of patients, *p* = 0.0016), while oral semaglutide was recurrently used in case of failure of previous treatment (66% versus 42.9% of patients, *p* = 0.0001), and especially to replace a previous treatment regimen including a DPP4-inhibitor (26.95% versus 11.8% of patients, *p* = 0.0016) or a sulphonylureas (16.5% versus 6.2% of patients, *p* = 0.0057) or pioglitazone (3.5% versus 0% of patients, *p* = 0.012) (Fig. 1). Subcutaneous semaglutide, on the other hand, was more frequently used to replace a previous regimen including an insulin (prandial or basal or both), compared with oral semaglutide (10.2% and 4.2% of cases, respectively), but the difference did not reach statistical significance.

In either group, when used in dual therapy, the most frequent drug associated with semaglutide was metformin; in two cases oral semaglutide was prescribed in addition to SGLT2-I, and in two cases subcutaneous semaglutide was added to basal insulin.

In triple therapy, oral semaglutide was more often prescribed in addition to metformin and SGLT2i than subcutaneous semaglutide (6.95% versus 2.2%; *p* = 0.048), while once weekly semaglutide was preferentially added to a treatment regimen with metformin and basal insulin than oral semaglutide (9% versus 1.75%; *p* = 0.01).

After replacement of previous drugs, either oral or subcutaneous semaglutide were mostly associated to metformin in dual therapy (43.5% and 46.1%, respectively), followed by the association to metformin and basal insulin (14.5% and 18.5%, respectively), without significant differences between the two formulations. In 17.1% of the cases, oral semaglutide was combined with metformin and SGLT2i, while subcutaneous semaglutide was used in such combination therapy in only 1 case (1.3%) (*p* = 0.0008). In contrast, OW semaglutide was more commonly combined with metformin and pioglitazone than the oral formulation (9.2% vs. 1.3%, respectively; *p* = 0.029), when replacing other previously used drugs. A detailed description of treatment schedule is provided in Table S1.

### Treatment outcomes in T2D patients treated with semaglutide

Follow-up data were available in 130 patients treated with OW subcutaneous semaglutide and 81 patients treated with oral semaglutide. Patient characteristics are shown in Table 2. In our cohort of patients, 6 months treatment with semaglutide, either oral or subcutaneous, induced an overall improvement of metabolic control.

**Table 2:** Anthropometric measures and metabolic data of diabetic patients treated with oral or subcutaneous semaglutide, after 6 months of therapy

	Oral Semaglutide (n = 81)	*p* value vs T0	OW Semaglutide (n = 130)	*p* value vs T0	*p* value oral vs OW
BW (kg)	78.5 ± 15.7 (48–125)	**0.0005**	93.7 ± 22.2 (45–158)	** < 0.0001**	** < 0.0001**
%WL	− 2.1 ± 7.6 (− 20.59–42.6)	n.a	− 6.2 ± 5.8 (− 25.7–12.3)	n.a	< 0.0001
BMI (kg/m^2^)	28.4 ± 4.9 (19.5–41.1)	** < 0.0001**	33.0 ± 6.9 (21–58)	** < 0.0001**	** < 0.0001**
% BMI loss	− 2.7 ± 4.6 (− 20.6–7.2)	n.a	− 6.3 ± 5.5 (− 25.6–6.7)	n.a	** < 0.0001**
WC	102.9 ± 12.8 (86–143)	**0.008**	114.2 ± 17.2 (86–157)	** < 0.0001**	**0.007**
% WC loss	− 3.7 ± 6.1 (− 14.3–7.8)	n.a	− 6.1 ± 3.9 (-14.8–1.3)	n.a	0.06
A1c (%)	7.4 ± 1.9 (5.2–18.4)	0.1 (**)	6.6 ± 0.9 (5.3–9.8)	** < 0.0001**	**0.0001**
Delta A1c	− 0.4 ± 2.3 (− 6.9–11.7) (*)	n.a	− 1.1 ± 1.4 (− 6.0–1.3)	n.a	**0.0007** (#)
FPG (mg/dL)	140.0 ± 63.9 (70–556)	0.5 (**)	118.5 ± 32.4 (64–195)	** < 0.0001**	**0.001**
Total cholesterol (mg/dL)	162.6 ± 39.9 (93–267)	**0.04**	159.9 ± 42.5 (78–272)	** < 0.0001**	0.5
Triglycerides (mg/dL)	139.8 ± 58.9 (33–324)	**0.02**	139.4 ± 80.4 (50–720)	0.09	0.4
HDL (mg/dL)	49.2 ± 13.6 (26–107)	0.1	48.1 ± 12.1 (24–86)	0.4	0.8
LDL (mg/dL)	85.8 ± 37.9 (10.4–193)	**0.007**	84.4 ± 35.7 (13.4–175.4)	** < 0.0001**	0.7
Creatinine (mg/dL)	0.94 ± 0.32 (0.37–2.04)	0.7	0.97 ± 0.33 (0.50–2.56)	**0.01**	0.4
eGFR (mL/min/1.73 m^2^)	80.0 ± 20.9 (32.0–117.9)	0.5	78.3 ± 23.2 (18–131)	**0.006**	0.8
Albuminuria (mg/dL)	34.8 ± 131.1 (0–982)	0.38	31.8 ± 86.7 (0–696)	0.85	0.9
ACR (mg/g)	8.196 ± 20.2 (0–82.5)	0.57	2.05 ± 4.5 (0–24.8)	0.14	0.3
AST (UI/L)	21.1 ± 7.3 (11–47)	0.55	18.1 ± 6.3 (7–39)	**0.004**	**0.03**
ALT (UI/L)	20.9 ± 9.8 (5–46)	0.19	19.0 ± 9.1 (4–50)	**0.0008**	0.4
GGT (UI/L)	25.7 ± 17.4 (4–86)	0.19	27.2 ± 24.9 (8–146)	0.83	1.0
Uric acid	5.6 ± 1.3 (2.9–7.3)	**0.04**	5.7 ± 1.3 (3.8–9.0)	0.79	0.8
SBP	135.5 ± 19.7 (95–175)	0.74	137.0 ± 19.6 (90–190)	**0.002**	0.6
DBP	76.4 ± 8.9 (58–90)	0.66	79.7 ± 11.0 (55–106)	0.50	0.1

Data are expressed as mean ± SD, range is shown in brackets. *BMI* body mass index; *BW* body weight; *WC* waist circumference; *%WL* percentage of weight loss; *%EWL* percentage of excess weight loss; *A1c* glycated hemoglobin; *FPG* fasting plasma glucose; *HDL *high density lipoprotein; *LDL* low density lipoprotein; *eGFR* estimated glomerular filtration rate, calculated by MDRD (Modification of Diet in Renal Disease) equation; *ACR* albumin-to-creatinine ratio; alanine transaminase (ALT); aspartate transaminase (AST); gamma-glutamyl transpeptidase (GGT); *SBP* systolic blood pressure; *DBP* diastolic blood pressure. Statistics using non-parametric Mann–Whitney U test for comparison between different groups and Paired t-Test for comparison between T0 and T6 parameters in the same group

(*) −0.5 ± 1.9 (−6.9–4.8) when excluding the outsider value

(**) *p* = 0.01 and p = 0.05 respectively when excluding the outsider value

(#) *p* = 0.001 when excluding the outsider value

**Fig. 2:**
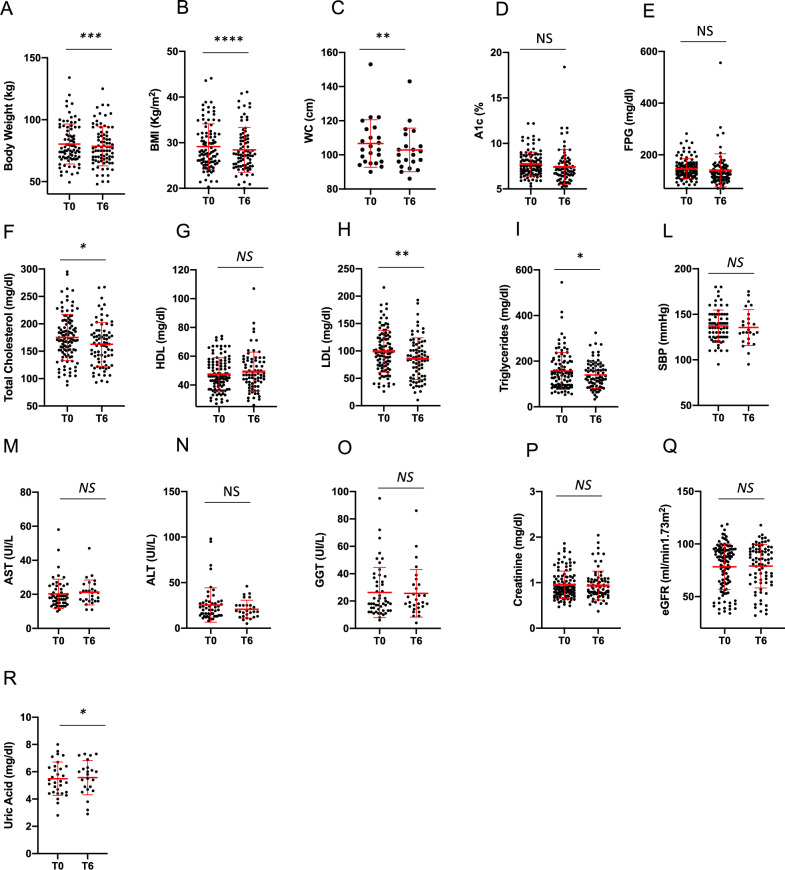
Comparison of anthropometric (body weight **(A)**, BMI **(B)**, WC **(C)**) and metabolic parameters (A1c **(D)**, FPG **(E)**, total cholesterol **(F)**, HDL cholesterol **(G)**, LDL cholesterol **(H)**, triglycerides **(I)**, creatinine **(L)**, eGFR **(M)**, uric acid **(N)**, AST **(O)**, ALT **(P)**, GGT **(Q)**) and systolic **(R)** and diastolic **(S)** blood pressure between baseline (T0) and 6 months (T6) in oral semaglutide group. NS = not significant; A1c: glycated hemoglobin; FPG: fasting plasma glucose; HDL: high density lipoprotein; LDL: low density lipoprotein; eGFR: estimated glomerular filtration rate, calculated by MDRD (Modification of Diet in Renal Disease) equation; alanine transaminase (ALT); aspartate transaminase (AST); gamma-glutamyl transpeptidase (GGT). *p < 0.05, **p < 0.01, ***p < 0.0001 by Paired T-test test. Error bars are shown as mean ± SD

Patients in the oral semaglutide group experienced a reduction in BW, BMI and WC (respectively *p* = 0.0005, *p* < 0.0001 and *p* = 0.008) (Fig. 2a–c). We also observed a non-significant reduction of A1c and FPG after 6 months of therapy (Fig. 2d, e). However it is worth noting that one patient presented with a severe hyperglycemia at follow-up (A1c = 18.4%); when excluding this patient from the analysis, A1c reduction from baseline (− 0.5 ± 1.9) proved significant (*p* = 0.01). Patients also experienced a significant reduction of total and LDL cholesterol levels and triglycerides (respectively, *p* = 0.04, *p* = 0.007 and *p* = 0.02) (Fig. 2f, h, i). Additionally, a slight, although significant, increase in uric acid levels was observed (*p* = 0.04) (Fig. 2r).

**Fig. 3:**
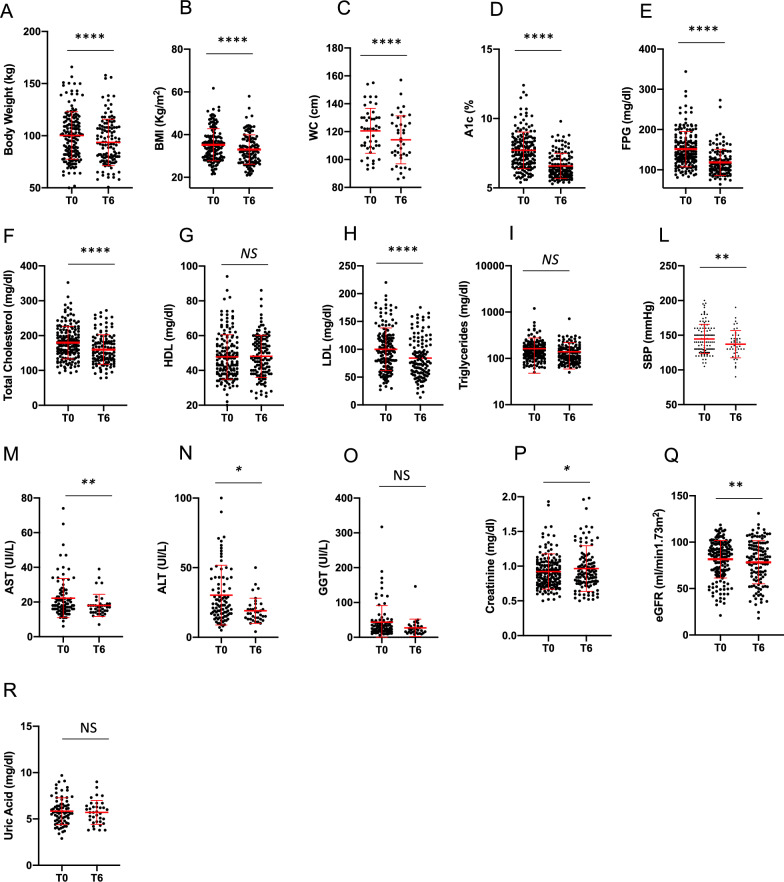
Comparison of anthropometric (body weight **(A)**, BMI **(B)**, WC **(C)**) and metabolic parameters (A1c **(D)**, FPG **(E)**, total cholesterol **(F)**, HDL cholesterol **(G)**, LDL cholesterol **(H)**, triglycerides **(I)**, creatinine **(L)**, eGFR **(M)**, uric acid **(N)**, AST **(O)**, ALT **(P)**, GGT **(Q)**) and systolic **(R)** and diastolic **(S)** blood pressure between baseline (T0) and 6 months (T6) in subcutaneous semaglutide group. NS = not significant; A1c: glycated hemoglobin; FPG: fasting plasma glucose; HDL: high density lipoprotein; LDL: low density lipoprotein; eGFR: estimated glomerular filtration rate, calculated by MDRD (Modification of Diet in Renal Disease) equation; alanine transaminase (ALT); aspartate transaminase (AST); gamma-glutamyl transpeptidase (GGT). *p < 0.05, **p < 0.01, ***p < 0.0001 by Paired T-test test. Error bars are shown as mean ± SD

OW subcutaneous semaglutide proved effective in significantly reducing BW, BMI and WC (*p* < 0.0001 for all parameters) and we also observed a significant reduction in A1c and FPG (*p* < 0.0001 for both parameters) (Fig. 3a–e). A significant reduction in total and LDL cholesterol levels was also evident at T6 (*p* < 0.0001 for both parameters), while the reduction in triglycerides levels did not reach statistical significance (*p* = 0.07) (Fig. 3f, h, i). Patients also experienced a significant reduction in systolic blood pressure (*p* = 0.002) and hepatic enzymes alanine transaminase (ALT) and aspartate transaminase (AST) (respectively, *p* = 0.0008 and *p* = 0.004), consistently with the significant weight loss obtained (Fig. 3l–n). We also found an increase in creatinine levels (*p* = 0.01) with slight—though significant—reduction in GFR estimated by the CKD-EPI formula (*p* = 0.006), of uncertain interpretation (Fig. 3p, q).

**Fig. 4:**
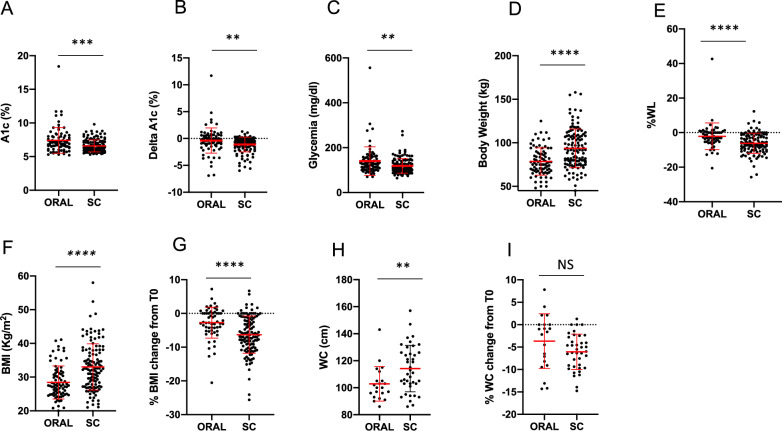
Comparison of metabolic data (A1c **(A)**, A1c change from baseline **(B)**, FPG **(C)**) and anthropometric measures (weight loss **(D)**, BMI **(E)** and WC **(F)** change from baseline, body weight **(G)**, BMI **(H)**, WC **(I)**) between oral and subcutaneous semaglutide group after 6 months of therapy. NS = not significant; A1c: glycated hemoglobin; FPG: fasting plasma glucose; WL: weight loss; BMI: body mass index; WC: waist circumference. *p < 0.05, **p < 0.01, ***p < 0.0001 by Mann–Whitney test. Error bars are shown as mean ± SD

When comparing the two different formulations, OW subcutaneous semaglutide seemed to be more effective than oral formulation on metabolic and anthropometric measures after 6 months (Table 2; Fig. 4a–i). Subcutaneous semaglutide induced a greater A1c reduction from baseline and a significant greater reduction of BW and BMI, although patients in the OW semaglutide group still displayed a higher BW, BMI and WC at T6 (Fig. 4a–i). On average, we observed an average reduction of 0.6 kg and 5.5 kg with oral and subcutaneous semaglutide, respectively. Concerning glycemic control, after excluding the patient with severe hyperglycemia on oral semaglutide, delta A1c from baseline was still significantly higher with injectable (− 1.1 ± 1.3) than with oral (− 0.5 ± 1.9) formulation. The effects on lipid profile and renal function did not differ significantly between the two groups, as well as on blood pressure (Supplementary Fig. S2a–g, m, n). Only patients treated with OW semaglutide showed a reduction of AST levels at T6, which resulted significantly lower than in patients taking the oral formulation (*p* = 0.04) (Supplementary Fig. S2h).

During follow-up, 12 patients in the oral semaglutide (10.4%) and 11 patients in the subcutaneous semaglutide (6.2%%) group discontinued treatment due to side effects, without significant differences between the two formulations (*p* = 0.27) (Supplementry Figure S2o); other less frequent causes of discontinuation were patients’ choice, therapeutic failure or drug shortage—only for subcutaneous formulation. The most frequently reported side effects were mild to moderate gastrointestinal events such as nausea, vomiting, constipation, diarrhoea or bloating, as expected. Of note, almost 15% of patients were lost to follow-up in both groups (*p* = 0.7, data not shown).

## Discussion

The high efficacy and safety of semaglutide make it an advantageous choice of T2D treatment, either subcutaneously and orally. In 2020, oral semaglutide has been selected as one of the drugs, which represent significant progress and outstanding contribution to public health in providing patients with another option to treat diabetes without injections [22].

Aim of our study was to assess and further clarify the use and different prescriptive profile of oral semaglutide compared to injective formulation in a real-world setting.

In our cohort, patients starting treatment with oral semaglutide had lower weight and BMI, but were older than those included in the OW semaglutide group, who, by contrast, already had obesity-related complications (hypertension, steatosis, albuminuria) at baseline, albeit with a shorter disease duration.

Regarding oral semaglutide, in contrast to what was shown in a recent Italian retrospective study, it was mainly prescribed in patients with a disease duration of more than 10 years [23]**.**

The higher efficacy in weight reduction demonstrated in clinical trials in early, established or advanced TD2 versus placebo or active comparators [24] might influenced the choice of OW semaglutide in higher class of obesity. However, considering the baseline characteristics of the population enrolled in the SUSTAIN and PIONEER programs, BMI in our population was still higher in OW semaglutide group and lower in oral semaglutide group [25, 26].

In both groups the mean age and disease’s duration resulted higher than the overall population of the main RCT studies [25, 26], which could derive from an older diabetic population in Italy than in other countries [27].

Recently, another Italian real-world retrospective study showed the efficacy and safety of oral semaglutide in a T2DM population, that was older compared with the PIONEER trials, similarly to the population described in our study [28].

Although initial glycemic control was similar between the two populations, subcutaneous OW semaglutide was more frequently prescribed as an intensification of therapy than oral semaglutide, and it was more commonly used in add-on to metformin. This finding may be justified by the need to achieve greater weight loss given the difference in the prevalence of obesity in the two groups [29]. On the contrary, oral semaglutide was more commonly used in place of a previous treatment regimen that especially included a DPP4-inhibitor or a sulphonylurea. Recent studies have demonstrated that switching from DPP4-inhibitor to oral semaglutide helps achieve HbA1c targets with less use of additional glucose-lowering medication and offers the potential for a greater reduction in BW, despite a slight risk of gastrointestinal symptoms [30, 31]. The switch from sulphonylurea is most likely due to the need to replace drugs associated to a greater risk of hypoglycemia and weight gain [5] with a more effective and safer alternative, which allows oral administration to be maintained [28, 32]. Of note, the percentage of patients treated with SGLT2 inhibitors is very low in our cohorts, but this finding is likely due to prescription limitations for the SGLT2i/GLP1-RA combination according to the Italian drug agency.

It is well-known that several treatment-related characteristics, including mode, timing, frequency of administration and complexity of the treatment, play a role in patients' preference and compliance. Many studies have specifically addressed factors influencing patient preferences, as this aspect is critical in improving adherence and reducing clinical inertia. No major difference in patient’s preference between the oral or injective forms has been shown in recent reports from different populations from UK and USA, so far [33, 34]. Even in our experience we didn’t find a significant difference in preference between the two drugs, though we do not have sufficient data in this regard. However, in studies showing a preference for the subcutaneous formulation, patients’ choice was mainly driven by the greater convenience of a weekly injection rather than a daily (albeit oral) treatment, which needed binding requirements (i.e. taking the tablet “on an empty stomach when you first wake up”, “with a sip of plain water”, and “wait at least 30 min after taking this tablet before eating, drinking, or taking other oral medications”) [35].

As expected, after 6-months follow-up, our data demonstrate an improvement in glycemic control and BMI for both formulations, consistent with the efficacy reported in the literature. An indirect comparison between OW and oral semaglutide, provided by Alhindi et al. demonstrated that the former appears to have a slightly greater effect on HbA1c and body weight with increase in the incidence of gastrointestinal AEs. Nonetheless, clinically significant reductions in A1c and BW were observed with semaglutide, regardless of the method of administration [36].

In our cohort, OW seemed to be more effective on anthropometric and metabolic parameters, but the baseline differences within the two groups might have influenced the results and should be taken with caution.

In the United States, a retrospective analysis using medical and pharmacy claims data between 2018 and 2020 from commercial and Medicare Advantage with Part D insurance shows a mean A1c change of − 0.8%. Among a subset of patients with A1c ≥ 9%, the mean A1c change was − 2.7% [37]. In our cohort, oral semaglutide showed a mean A1c reduction of − 0.5%, similar to previous reports, while a greater reduction was observed with subcutaneous semaglutide (mean A1c reduction of − 1.1%); in subjects with baseline A1c ≥ 9%, (respectively, 16 in the oral semaglutide and 25 in the subcutaneous semaglutide group) the delta A1c was –3.3% for both formulations (*p* = 0.9, data not shown), consistently with previous findings of greater efficacy in patients with higher baseline A1c values.

Exposure–response analyses showed a greater magnitude of reduction in A1c and BW as drug exposure increased; greater variability in plasma concentrations of oral semaglutide was also observed, with a wider range of exposure than observed with the subcutaneous formulation. However, the study reported considerable overlap in the plasma concentrations achieved with the different dosages of both formulations. Thus, the study authors concluded that the exposure–response relationship, both in terms of efficacy and tolerability, were similar regardless of the route of semaglutide administration [24]. In our cohort, we observed that most patients in the oral semaglutide group had not reached the maximum dose at the time of follow-up, which may have influenced the lower reported efficacy compared with the weekly formulation.

An improvement in lipid profile was also observed in both formulations, with significant reduction in LDL. Patients treated with OW semaglutide showed greater reductions in transaminases values than patients receiving oral semaglutide. A study by Newsome et al., designed to evaluate semaglutide as a potential treatment for Non-alcoholic Fatty Liver Disease (NAFLD)/Non-alcoholic Steatohepatitis (NASH), showed a dose dependent decrease in ALT levels, with maximal reductions occurring around week 28 and remaining stable thereafter [38]. Indeed, in our cohort, a slightly higher proportion of patients reached the maximal dose of subcutaneous semaglutide compared to oral semaglutide (27.5% versus 20.8%).

Both drugs were well tolerated: overall less than 10% of patients discontinued treatment due to the occurrence of side effects, with no significant differences between the two formulations, despite a slightly higher discontinuation rate in oral semaglutide group. As expected, the most frequently reported side effects were mild to moderate gastrointestinal events such as nausea, vomiting and diarrhea. This data appears to align with what emerges from the literature [39]. The occurrence of side effects is often dose-dependent, with higher doses associated with more frequent gastrointestinal events, but we have no data on this correlation (40). Moreover, we must point out that the appearance of side effects was recorded only in patients who actually suspended the treatment, and this may have led to an underestimation of their prevalence.

To our knowledge, this is the first study that analyzed in detail the prescribing patterns and characteristics that guide the choice toward one or another formulation of semaglutide in a real-world setting, in light of comprehensive clinical and metabolic data.

To conclude, in our experience injectable semaglutide seems to be preferred in patients with excess weight and shorter disease duration, while the oral formulation was used later and especially after therapeutic failure of previous therapies. Six-months follow-up data indicate similar tolerability and efficacy of both formulations, despite subcutaneous semaglutide demonstrated greater efficacy. However, further and larger studies are needed in order to distinguish a more specific prescribing profile for the two drugs, to compare their efficacy and safety over a longer follow-up period and to support clinical decision-making.

## Supplementary Information

Below is the link to the electronic supplementary material.

Supplementary file1 (PDF 592 KB)

Supplementary file2 (PDF 314 KB)

Supplementary file3 (PDF 65 KB)

## Data Availability

The datasets generated during and/or analyzed during the current study are available from the corresponding author on reasonable request.
