# Supplementary figures and images for: Real-world use of oral versus subcutaneous semaglutide in a cohort of type 2 diabetic patients: which option to which patient?
Source: J Endocrinol Invest. 2024 Apr 29;47(11):2679–90. doi: 10.1007/s40618-024-02369-4 (PMC11473455; doi:10.1007/s40618-024-02369-4)

Figure S1.

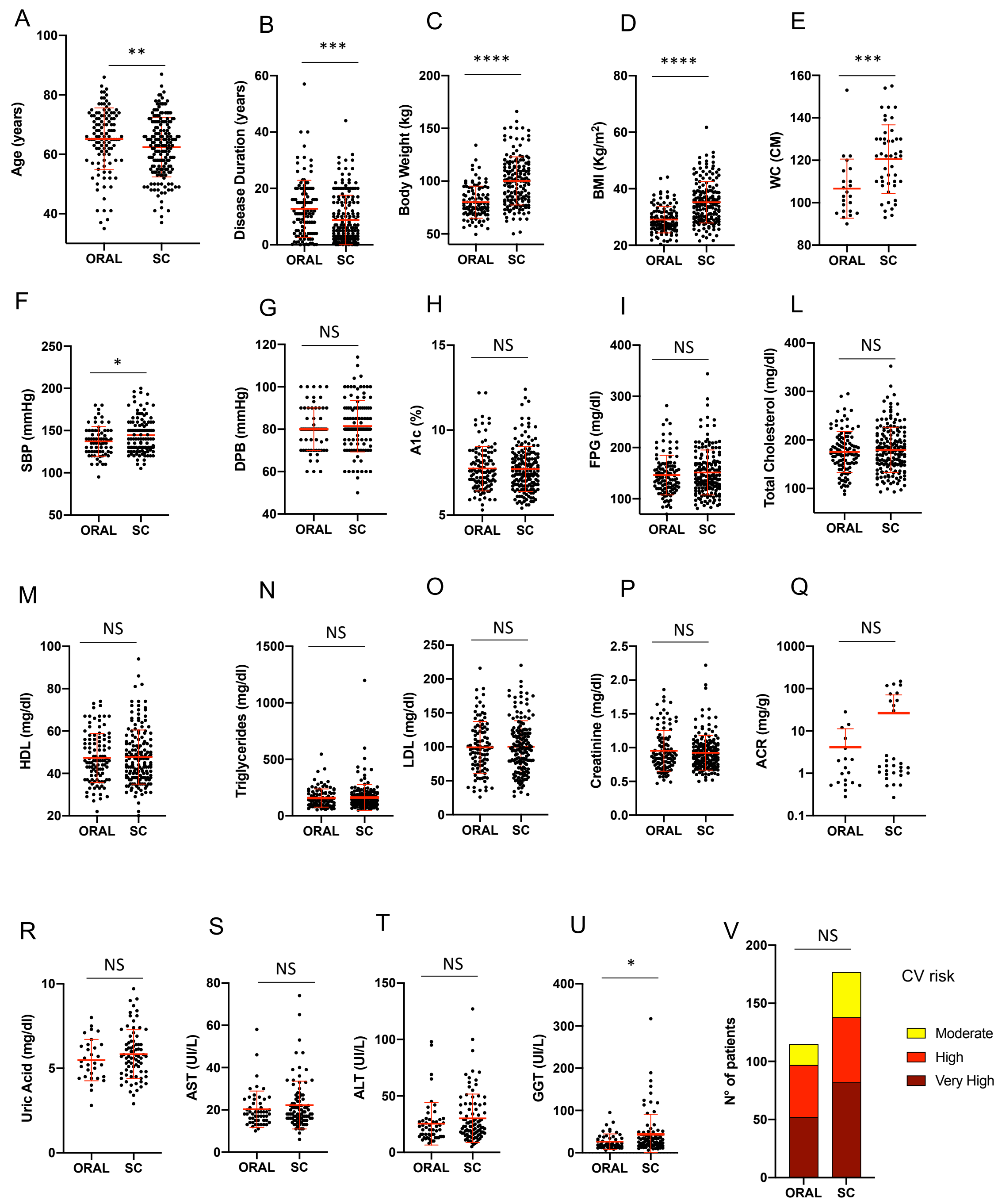

Supplement: Supplementary file 1 — Supplementary file1 (PDF 592 KB) [file 40618_2024_2369_MOESM1_ESM.pdf]

Figure S2.

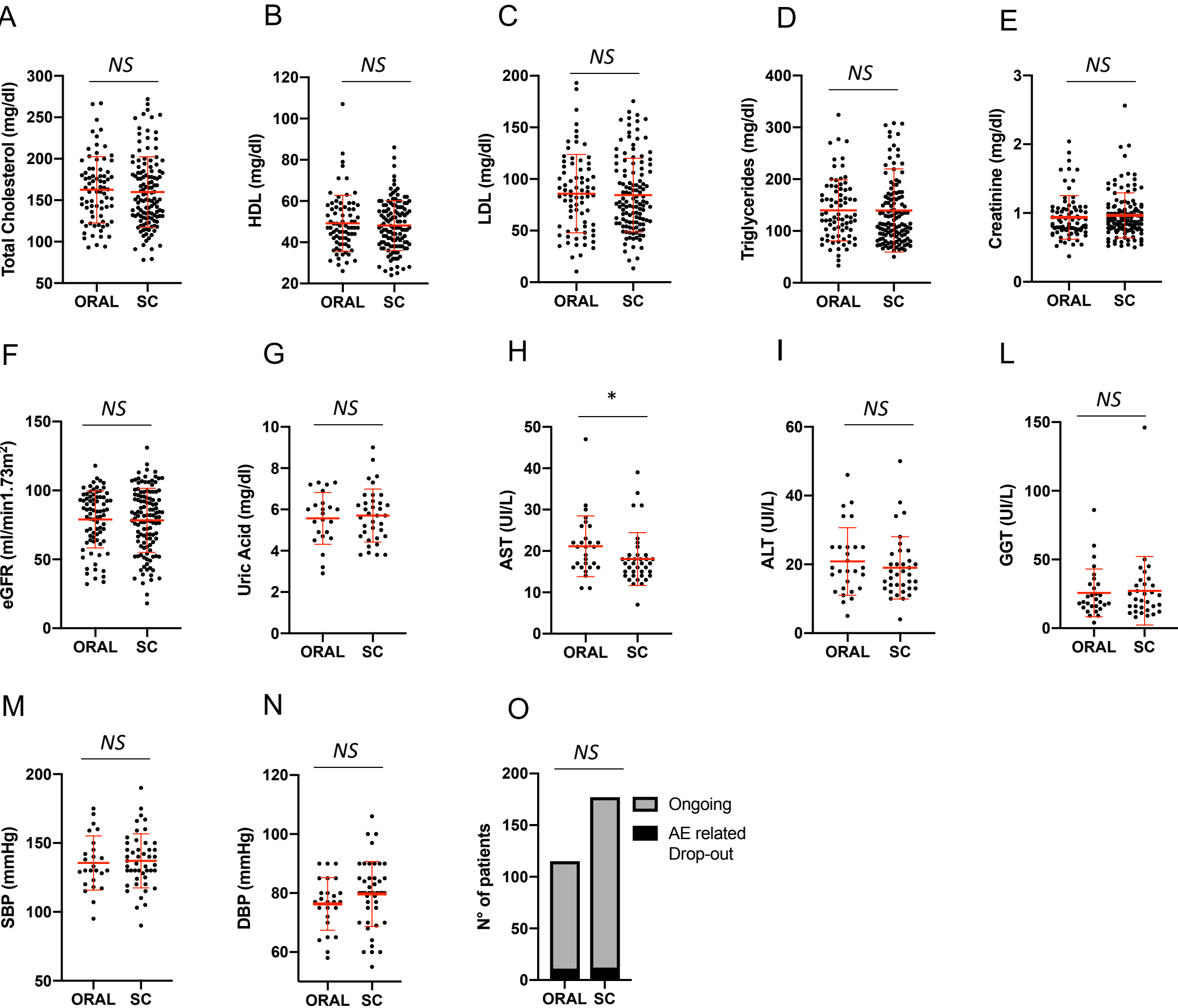

Supplement: Supplementary file 2 — Supplementary file2 (PDF 314 KB) [file 40618_2024_2369_MOESM2_ESM.pdf]
