# Supplementary material for: Real-world use of oral versus subcutaneous semaglutide in a cohort of type 2 diabetic patients: which option to which patient?
Source: J Endocrinol Invest. 2024 Apr 29;47(11):2679–90. doi: 10.1007/s40618-024-02369-4 (PMC11473455; doi:10.1007/s40618-024-02369-4)
Supplement: Supplementary file 3 — Supplementary file3 (PDF 65 KB) [file 40618_2024_2369_MOESM3_ESM.pdf]

Table S1.

Treatment schedule		Oral Semaglutide	OW Semaglutide	*p* value
**Monotherapy**		1 (0,9%)	1 (0,6%)	0,75
**Dual Therapy**		24 (20,9%)	67 (37,9%)	**0,0022**
	**Add-on to metformin**	22 (19,15%)	65 (36,7%)	**0,0016**
	**Add-on to basal insulin**	0	2 (1,2%)	0,25
	**Add-on to SGLT2-i**	2 (1,75%)	0	0,07
**Triple Therapy**		14 (12,2%)	33 (18,6%)	0,14
	**Add-on Met+Basal**	2 (1,75%)	16 (9%)	**0,011**
	**Add-on Met+SGLT2i**	8 (6,95%)	4 (2,2%)	**0,048**
	**Add-on Met+Pio**	2 (1,75%)	6 (3,4%)	0,4
	**Add-on Met+SUs**	2 (1,75%)	3 (1,7%)	0,9
	**Add-on BB**	0	1 (0,6%)	0,4
	**Add-on Met/Empa+Basal**	0	3 (1,7%)	0,16
**Replacement therapy**		76 (66%)	76 (42,9%)	**0,0001**
	**DPP4i (±*SGLT2i o SUs)***	31 (26,95%)	21 (11,8%)	**0,0016**
	**SUs**	19 (16,5%)	11 (6,2%)	**0,0057**
	**GLP1-RA**	9 (7,8%)	12 (6,7%)	0,7
	**SGLT2i**	8 (6,95%)	11 (6,2%)	0,8
	**Metformin**	0	2 (1,2%)	0,25
	**Pioglitazone**	4 (3,5%)	0	**0,012**
	**Acarbose**	0	1 (0,6%)	0,4
	**Insulin (basal and/or rapid analogues)**	5 (4,3%)	18 (10,2%)	0,07
**Post-replacement therapy (*)**	**+Metformin**	33 (43,5%)	35 (46,1%)	0,7
	**+Basal Insulin**	7 (9,2%)	5 (6,6%)	0,5
	**+Pioglitazone**	3 (3,9%)	0	0,08
	**+SGLT2i**	2 (2,6%)	0	0,15
	**+Met+Basal**	11 (14,5%)	14 (18,5%)	0,5
	**+Met+SGLT2i**	13 (17,1%)	1 (1,3%)	**0,0008**
	**+Met+Pio**	1 (1,3%)	7 (9,2%)	**0,029**
	**+Met+SUs**	0	3 (3,9%)	0,08
	**+SGLT2i+Basal**	0	3 (3,9%)	0,08
	**+Met+Pio+SGLT2i**	0	1 (1,3%)	0,3
	**Monotherapy**	6 (7,9%)	5 (6,6%)	0,7
	**+Met+SGLT2i+Basal**	0	1 (1,3%)	0,3
	**+ BB (±*Met*)**	0	1 (1,3%)	0,3

(*)Percentage calculated on the total number of patient who received od/ow semaglutide as replacement therapy
